# How mothers of terminally ill children cope and appraise their life situation

**DOI:** 10.1192/j.eurpsy.2022.606

**Published:** 2022-09-01

**Authors:** E. Bityutskaya, N. Lebedeva

**Affiliations:** 1 Lomonosov Moscow State University, Faculty Of Psychology, Moscow, Russian Federation; 2 Moscow Metropolitan Governance University, Diagnostics Department, Moscow, Russian Federation

**Keywords:** life situation, coping strategies, subjective appraisal

## Abstract

**Introduction:**

Mothers of terminally ill children experience chronic stress that can lead to physical and emotional exhaustion. A better understanding of their experiences, resources, and vulnerabilities can help plan psychological interventions.

**Objectives:**

The research is aimed to study mothers’ appraisals of their life situation related to the child’s terminal illness and their coping strategies.

**Methods:**

Participants: 21 women (aged 26-49) whose children were patients at the hospice. Women answered a set of open-ended questions and completed questionnaires: “Appraisal Criteria of the Difficulty of a Life Situation”, “Types of Orientations in Difficult Situation”, “Ways of Coping Checklist”. Data were analyzed with Pearson’s r.

**Results:**

Planned coping was associated with evaluations of opportunity (r=0.78) and threat to the future (r=0.61). Despite the deteriorating health status of most of the children, women reported a high degree of subjective control. This might be related to outside help. Participants stated that helping by the family, doctors, and psychologists was essential. Unexpectedly, the “need for a quick and active response” score correlates with that for the coping strategy “fantasizing” (r=0.62). This can be explained by the depletion of resources; deprivation of sleep and active rest is often observed. An important feature is that half of the participants report high self-blame.

**Conclusions:**

We suggest that self-blame, an analysis of opportunities, and probable future scenarios are important psychotherapy targets for mothers of terminally ill children. The consequences of threat appraisal are twofold: admitting the threat can be painful, but it also mobilizes one’s energy. Funding: The study was funded by RFBR, project number 20-013-00838.

**Disclosure:**

No significant relationships.

